# AFT survival model to capture the rate of aging and age-specific mortality trajectories among first-allogeneic hematopoietic stem cells transplant patients

**DOI:** 10.1371/journal.pone.0193287

**Published:** 2018-03-02

**Authors:** Yuhui Lin

**Affiliations:** NaoRococo at The Waterhouse, Singapore, Singapore; Universiti Putra Malaysia, MALAYSIA

## Abstract

Accelerated failure time (AFT) model is commonly applied in engineering studies to address the failure rate of a machine. In humans, survival profile of transplant patients is among the rare scenarios whereby AFT is applicable. To date, it is uncertain whether reliable risk estimates and age-specific mortality trajectories have been published using conventional statistics approach. By investigating mortality trajectory, the rate of aging *d(log(μ(x)))/dx* of Hematopoietic Stem Cells Transplants (HSCTs) patients who had underwent first-allogeneic transplants can be obtained, and to unveil the possibility of elasticity of human aging rate in HSCTs. A modified parametric frailty survival model was introduced to the survival profiles of 11,160 patients who had underwent first-allogeneic HSCTs in the United States between 1995 and 2006; data was shared by Center for International Bone and Marrow Transplant Research. In comparison to stratification, the modification permits two entities in relation to time to be presented; age and calendar time. To consider its application in empirical studies, the data contains arbitrary right-censoring, a statistical condition which is preferred by choice in many transplant studies. The finalized multivariate AFT model was adjusted for clinical and demographic covariates, and age-specific mortality trajectories were presented by donor source and post-transplant time-lapse intervals. Two unexpected findings are presented: i) an inverse J-shaped hazard in unrelated donor-source *t≤100-day*; ii) convergence of unrelated-related hazard lines in *100-day<t ≤ 365-day* suggests maximum manifestation of senescence among survivors. Analyses of long-term survivors (*t>365-day*) must consider for periodic medical improvements, and transplant year as a standalone time-variable is not sufficient for statistical adjustment in the finalized multivariate model. In relevance to clinical studies, biennial event-history analysis and age-specific mortality trajectories of long-term survivors provide a more relevant intervention audit report for transplant protocols than the popular statistical presentation; *i*.*e*. survival probabilities among donor-source.

## Introduction

By 2050, approximately 15% of the world population will be aged 65 and older, and with the increasing proportion of the population living to older ages, clinicians and researchers have already observed and reported an increased incidence of age-related chronic diseases such as cardiovascular-related diseases, age-specific cancer-types and neurodegenerative diseases.[1–4] Organ transplants are in demand to not only offer patients a life-saving opportunity, but to also reduce the overall burden in public health. It is uncertain on whether the organ donor-recipient demands will ever be met, but a rigorous data analysis can offer insights into how this life-saving opportunity may alter the age-specific mortality trajectory driven by donor-source, and if extreme changes in the immunological system have the ability to alter the human rate of aging at each post-transplant time-lapse interval.

The classic parametric framework to study mortality dynamics of human populations uses the Gompertz hazard function; *μ(x)* = aebx,[5–9] and an assumed gamma-distributed frailty Gompertz function has been shown to provide a better fit for adulthood in longitudinal studies and in life-table analyses [10–13]; Panels A and B in S1 Fig. However, in transplant studies, the underlying baseline hazard shape may not follow the mathematical rules of the Gompertz function, and the shape of the hazard may change depending on time-lapse since transplant surgery. The assessment of post-transplant hazard shape requires a different parametric survival model that has the capability to accommodate flexibility in its hazard shape; *i*.*e*. creates hazard bends in accordance to follow-up survival time and to age at death. Herein, I present the analysis of a modified AFT survival regression of patients who had received their first allogeneic hematopoietic stem cells transplant (HSCT). Information on post-transplant HSCT patients longitudinal survival follow-up were recorded by the Center for International Blood and Marrow Transplant Research (CIBMTR) and their respective transplant centers in the United States. Hence, the data framework permits a survival regression analysis that considers observed heterogeneity commonly referred as clinical covariates *e*.*g*. acute and chronic Graft-versus Host Disease (GvHD), disease group, total body irradiation, related and unrelated donor-source, waiting time to first-allogeneic transplant, prior autologous transplant, morbidity score index, year of birth, gender-effects, transplant year, conditioning regimen and geographic regions in the United States. Further demographic details provided the basic requirements to perform a survival analysis; transplant age, age at death or failed transplant, vital status at last follow-up; Table 1. In contrast to published survival rates of transplant studies, *t<6-week* post-transplant survival was also considered as part of the studied population for an overview of its hazard shape.

**Table 1:** Characteristics of CIBMTR first allogeneic patients by donor source.

N_total_ = 11, 160	HLA-matched or related	Unrelated
N	3363	7797
Females %	42.5	43.2
Died %	64.4	71.3
Diagnosis to Transplant% <6mths	40.2	22.1
Acute GvHD (Grade II—IV)	34.3	43.1
Chronic GvHD	43.2	47.0
Acute duration (mean mths)*	14.9	19.2
Chronic duration (mean mths)*	24.4	24.0
Post-transplant Survival, Duration %		
≤100 days	23.1	28.3
100 days<t ≤365 days	23.3	23.8
>365 days	53.6	47.9
**Conditions prior to transplant %**		
Karnofsky score (≥80%)^	84.5	82.1
Prior autologous transplant	0.3	11.8
Myeloablative	78.2	71.5
Conditioning regimen%		
Bu+Cy+-Other	33.8	18.6
TBI+Cy+-Other	33.1	43.0
TBI+-Other (No Cy)	6.7	3.4
BU+-Other (No Cy)	3.2	4.8
TBI+Cy+Flud+-Other	0.4	0.2
TBI+Flud+-Other (No Cy)	3.4	6.2
Bu+Flud+-Other	3.5	5.9
Melphalan+Flud+-Other	3.2	7.6
Cy+Flud+-Other	4.3	3.1
Other	8.3	7.1
Total body irradiation (cGy)%		
No TBI	55.1	45.8
*≤400*	3.9	6.7
401–600	2.8	3.1
601–800	0.1	0.1
801–1000	3.5	3.4
1001–1200	20.8	18.5
>1200	13.7	22.5
TBI dose missing	0.1	0.0
Disease%		
ALL	9.2	11.5
AML	36.0	38.4
CML	21.8	22.2
Lymphoma	23.7	16.3
MDS	9.3	11.5
Graft-types%		
Bone marrow	30.8	54.2
Peripheral Blood + Bone Marrow	69.2	45.8

*: right-tailed distribution, but median does not serve inference purposes.

^: where unknown is < 10%.

Conditioning regimens abbreviations: TBI, total body irradiation; Cy, cyclophosphamide; Flud, fludarabine; Bu, busulfan; Mel, melphalan; FK506, tacrolimus; MMF, mycophenolate mofetil; MTX, methotrexate; CSA, cyclosporine

Disease: Acute Lymphoblastic Leukemia (ALL); Acute Myeloid Leukemia (AML); Chronic Myeloid Leukemia (CML); Myelodyplastic Syndromes (MDS).

Frailty, also known as unobserved heterogeneity has been considered to play a fundamental role to obtaining reliable risk estimates during survival analyses. However, a frailty distribution appears to be absent in this HSCTs population; log-normal-, gamma- and inverse gaussian- distributed frailty did not converge during the parametric univariate survival analyses of both conventional MLE survival approach and modified survival regression; S1–S3 Equations, and Equations 1.1–1.4.[11] The predominant reasoning is due to arbitrary right-censoring, a preset data condition which results in no measurable frailty during survival analysis and it limits the possibility of a Markov Chain and Monte-Carlo Markov Chain (MCMC) analysis. Although frailty component does not seem to serve any meaningful purpose in this study, it is important to recognize that allogeneic HSCT patients undergo a stringent selection for mortality as autologous transplant may not have been their possible life-saving option.

Though there are data limitations, this study shows: i) the age-specific mortality trajectories at each post-transplant time-lapse interval (*t*); ii) the rate of aging by donor-source and post transplant intervals; iii) the importance to adjust for medical improvements during survival analyses of HSCT long-term survivors; *t>365-day*.

## Methods

Estimation of the rate of aging requires the knowledge of the underlying hazard function, *i*.*e*. shape. Parametric models are therefore the only approach to obtain the rate of change of the hazard gradient, also known as the rate of aging or the relative derivative for mortality; *d(log(μ(x)))/dx*. According to post-transplant time-lapse intervals, proportion of GvHD inflammation risk-types, graft failure rates and mortality risk among patients can potentially vary across observational time. The two entities: calendar time (*tx*) and age (*x*, *j*) are determinants for the changes in the likelihood for mortality with the occurrence of continuous progress made in medicine and transplant protocols, and the rate of aging. Calendar time and age were both adjusted for during univariate and multivariate survival analyses; S1–S4 Tables.

### Survival analyses

Survival analyses require the following crucial elements for its likelihood estimation: i) age at death or censoring (*x*), ii) age at recruitment or transplant (*j*), iii) vital status of each individual at the end of follow-up (*θ*); S2 Fig. Based on previous radiation exposure-to leukemia studies, survival analyses were restricted to >30 years for ages at death and last follow-up was in 2012. As human aging is presumed to occur after the completion of growth and development, the exponential increase in mortality rate tends to occur after age 30; Panels A and B in S1 Fig. Recruitment age, also referred as transplant age, was restricted at below 70 years; **N.B.** transplant age is not equivalent to age-specific mortality trajectory. Patients whom had received allogeneic HSCs grafts were likely to experience a variation in mortality dynamics after successful transplant. Childhood and young adulthood cancer patients whom had benefited from HSCTs as a life-saving opportunity were likely to experience a different mortality dynamic to adult HSCT recipients, and such difference by development transition also occurs in the normal population but the parametric shapes have shown to be inconsistent; Panels A and B in S1 Fig. Thereby, this survival regression analysis would then serve to determine the mortality trajectory of transplant patients at aged>30, and an inadequate fit to the Gompertz-based survival model would then be suffice to prove that the conventional parametric approach is not suitable to addressing the mortality trajectory of first-allogeneic HSCT patients.

Patients with missing information on gender, transplant age, donor source, graft-type, conditioning regimen intensity and GvHD inflammatory responses were considered unreliable and were excluded for this analysis; *30*.*6%*. After the exclusion of missing information, patients were categorized as recipients of either unrelated- or related-HSCTs grafts (*N = 11*,*160*; Table 1). Human leukocyte antigen (HLA)-matched siblings, and other categorized related transplants were considered as related grafts. For unrelated-grafts, graft source contained well-matched, partially matched, mismatched and unknown degree matched. In consideration of the arbitrary right-censoring and the statistical power of the survival analyses, HSCT patients were grouped according to related/unrelated grafts received and the following three post-transplant survival time-lapse intervals: *t≤100-day; 100-day<t≤365-day and t>365-day*.

### MLE framework

The conventional MLE survival analysis framework of individual-based survival profiles is highly similar to the structure of actuarial life-tables. All individuals in the observational study must enter the MLE framework to obtain an accurate measurement of the event of interest which is a binary outcome; died or alive.

To achieve the general MLE structure, a suitable underlying hazard shape must be first determined. From the determined *h(x)*, the survival function *S(x)* is thereby deduced using S3 Equation. As patients had to be diagnosed for disease prior to transplant, the assumed survival function at transplant would be represented as *S(j)*.

**Equation 1. Hazard function**. Weibull hazard function is illustrated in this section as it is the most appropriate model for the parametric survival analysis of this specified HSCTs data. When the underlying hazard function has yet to be determined, a range of parametric models is fitted to the survival profiles and Aikake Information Criterion (AIC) scores assist in determining the most appropriate model.

**Equation 1.1. Individual-based Weibull hazard**

$$h(x)=\lambda kx^{\left(k-1\right)}$$

**Equation 1.2. Population-based hazard** with covariate represented as beta coefficient, θ as event indicator or right-censoring; dead = = 1 and alive = = 0. The power indicator specifies patients whom had experienced the event to contribute to the population hazard. δ is the binary matrices for post-transplant time-interval and donor-source which permits all individuals to enter the function and MLE. Therefore, when the values of δ matrix for all individuals is set as 1, the modified likelihood estimation returns to the conventional likelihood approach.

$$u(x)={\left[{\left(h(x)e^{\left(\beta Ζ\right)}\right)}^{\theta }\right]}^{\delta }$$

**Equation 2. Survival function**. Patients upon receiving life-saving opportunity must be alive to contribute to the observed variables, and exit at age at death or arbitrary right-censoring; x. Baseline hazard: Weibull.

$$S(x)={\left[(e^{-\lambda x^{k}})e^{\left(\beta Ζ\right)}\right]}^{\delta }$$

**Equation 3. Likelihood contribution of an individual’s profile**. The contribution of a patient’s survival profile to the MLE framework is therefore, Equation 1.2 and Equation 2. ω represents the two parameters of the Weibull function to be optimized, and j as the recruitment age for left truncation.

L(ω;x,j)=μ(x)+S(x)−S(j)

However, the summation of the three contributing elements of each individual may prove to be tedious especially in population-based studies whereby thousands and millions of individuals are involved. Hence, the *Laplace* transform is a useful approach for computation analysis; Equations 4.1 and 4.2.

**Equation 4.1. Survival function for left-truncation** whereby recruitment age is age at transplant. Patients must be alive to receive the life-saving opportunity and the assumed survival function is therefore the inverse of S(x) but replacing x for recruitment age, j.

$$tr.S(j)={\left[\frac{1}{S(j)}\right]}^{\delta }$$

**Equation 4.2. Log-Likelihood function**. The log-likelihood accounting for right-censoring, left-truncation. ω represents the two parameters of the Weibull function to be optimized.

$$\text{log}\;L(\omega ;x,j)=\sum_{}^{}\text{log}(\mu (x)S(x)tr.S(j))$$

The changes in the probability to developing GvHD and graft-rejection with increasing post-transplant survival time were also considered in the survival analysis. A logical matrix (δ) was then introduced to the parametric MLE framework refer to Equations 1.2–4. A further assumption was made during the analyses that all continuous periodic mortality progress *i*.*e* the availability of novel immunosuppression drugs and improvements made in transplant protocols would be captured during the biennial event history analysis (EHA) for calendar time 1995 to 2012, and post-transplant survivors *t>365-day* would have survived long enough to benefit made from medical progress. Long-term survivors *t>365-day* were categorized as a group as the right-tailed distribution presented in Supplementary information S3 Fig suggests that the statistical power for a parametric survival analysis would not be adequate for further multivariate analyses by categorical post-transplant time-lapse intervals; *t>24-months; t> 36-months*, etc. Furthermore, more than 80% of the patients died or experienced failed first allogeneic graft within *18 months* of post-transplant. More importantly, a separate *t>18-month* parametric survival analysis presented non-convergence during MLE and existing optimization methods were inconclusive. According to the three categories of post-transplant survival, survival status for each long-term survivor was traced at each biennial follow-up during EHA analysis and estimates were optimized using MLE; method ‘*SANN*’. All estimated parameters reached convergence. The standard errors and 95% confidence interval of the estimates were obtained using the ‘*delta*’ method from the *Hessian* matrix. As MLE is sensitive to the initial parameters set for optimization, 1,600 sets of initial parameters were used for each selection and the output of the best combination was presented in this manuscript. All statistical analyses and algorithms were written using *R-software version 3*.*0*.*3*.

### Model selections and multivariate analyses

**Fig 1:**
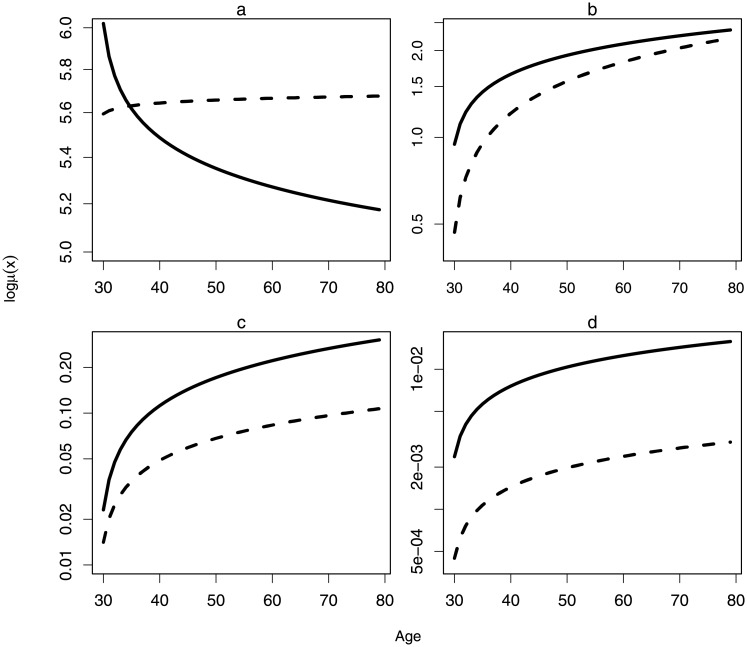
Age-specific mortality trajectories for first allogeneic HSCT patients by donor source. Weibull baseline hazard adjusted for covariates: *a) ≤ 100 days; b) 100-day <t ≤ 365-day; c) t>365-day* no EHA; *d) t>365-day* with EHA. Solid line: unrelated transplant; Dashed line: related transplant. *N*.*B*. *The y-axes expressed in semi-logarithmic scale*. Parameter estimates based on S4 Table in supplementary materials.

The most suitable parametric model was defined during the univariate analyses and was selected using Aikake Information Criterion (AIC), S1 Table. Parametric models: Gompertz, Gompertz-Makeham, Weibull, Weibull-Makeham, and a frailty component for unobserved heterogeneity was considered in the aforementioned models during the univariate analyses. Covariates listed in Table 1 were then introduced to the most suitable parametric model, and estimates by donor-source and post-transplant survival intervals were further deduced from the logical matrix in the finalized multivariate model. Multivariate analyses were achieved using forward-selection process of covariates to the survival model (S2 Table; significant covariates were listed in Finalized Model I), and comparison of a better-fit model was achieved using likelihood ratio test; *p-value<0*.*05* for statistical significance. Parameter estimates by donor sources were shown in S3 Table. As life-expectancy in the United States varies across states, the finalized model was then further adjusted for geographic regions; Supplementary information S4 Table and Fig 1.

In conjunction to the multivariate parametric frailty analysis, a semi-parametric Cox analysis was conducted to observe for changes in the magnitude for mortality risk and to consider the feasibility of a shared frailty by geographic regions in a data that was subjected to an arbitrary right-censoring, also known as first landmark in medical statistics. *N*.*B*. *Semi-parametric analysis commonly referred as the Cox model is not shown in this article as it is not applicable to the research hypothesis; no hazard shape for the assessment of the rate of aging*.

### Data characteristics

Center for International Blood and Marrow Transplant Research (CIBMTR) was established in 2004 for international collaborative work on transplant outcomes. The specified CIBMTR data consists of 159 transplant centers in the United States, and a total of 40 participating countries. Participating centers were required to report all transplants consecutively. Detailed data on hematopoietic stem cells transplantations are brought forward to a Statistical Center at the Medical College of Wisconsin in Milwaukee and the National Marrow Donor Program (NMDP). Compliance is monitored by on-site audits.

In this study, HSCTs patients in the United States diagnosed with acute myeloid leukemia, acute lymphoblastic leukemia, chronic myelogenous leukemia or lymphoma underwent first allogeneic transplant including patients with prior autologous transplant reported to CIBMTR between year 1995 and 2006 were considered during the survival analyses. Centers whose completeness follow-up index <80% at 5 years post-transplantation were excluded. Regions in the United States participated in this study: New England, Mid Atlantic, South Atlantic, East North Central, East South Central, West North Central, West South Central, Mountain, Pacific. Information on patients’ clinical treatments were recorded before and after transplant, and post-transplant survival status were updated at 100 days and six months, and annually thereafter.

This CIBMTR survival data had been previously analyzed and published by researchers to address different medical research hypotheses. The initial research hypothesis of this study was to estimate unobserved heterogeneity among HSCT patients and to estimate the changes in their rate of aging by donor source and time-lapse since transplant.

## Results

Post-transplant survival probability should be heterogeneous-mixed among transplant patients. The modified likelihood survival model permits the inclusion of demographic and transplant covariates, and matrices; pre-specified post-transplant time-lapse intervals and, related and unrelated donor-source; Equations 1–4. The post-transplant survival duration intervals, *t≤100-day; 100-day<t≤365-day*, *t>365-day* were pre-specified with an interest for changes in the rate of aging and age-specific mortality trajectories (S1 Fig). Univariate and multivariate models selection processes were achieved using the scores from Aikake Information Criterion (AIC—for parametric shape) and likelihood ratio test for finalized multivariate model; S1–S4 Tables. Based on AIC scores, Weibull model appears to be the best fitted model of HSCTs first allogeneic transplant patients. In HSCTs, the anchoring of new HSCs takes approximately six weeks and during this post-transplant interval, patients are highly susceptible to infectious diseases. In this study, deaths in the first six weeks accounted for 47.0% deaths of *t≤100-day* survival profiles (S2 Fig).

Most longitudinal transplant data analyses select patients whom had survived *t>6weeks* or *t>100-day* for survival or logistic regression analyses [14–16]. However, this analysis has taken an interest to also present the mortality dynamics of these short-term survivors and were expressed as age-specific mortality trajectory by donor-source and under the influence of acute GvHD onset; *t≤100-day* and *t>100-day*. HSCTs patients with post-transplant survival of *t>365-day* must have been subjected to receiving medical improvements with increasing survival time, and have also survived relatively longer to benefit from medical progress Fig 1. Clinical and demographic variables were also considered during multivariate analyses; Table 1 for descriptive analysis, and S1–S4 Tables for multivariate selection and optimized parameter estimates.

In all post-transplant intervals, patients whom had received unrelated-graft presented a higher mortality rate than related-graft transplants Panel A in Fig 1a. However, the age-specific mortality trajectories present an unexpected finding at *t≤100-day*: unrelated-HSCT presented inverse J-shaped and related-HSCT presented negligible senescence, also known as a flat hazard. Though unrelated-HSCT risk for mortality starts at a higher magnitude, the rate of aging decelerates with increasing age, *k* = 0.96. A hypothetical answer to this phenomenon is that the human immunological responses decline with increasing age, and though it is a phenomenon which is not favorable to combat against infectious diseases, a lower immune-reactivity gives a better graft tolerance. On the contrary, adults between aged 30 and 50 may have a better immune-reactivity, but are more likely to experience aggressive graft-rejection. Negligible senescence presented in related-HSCT *t≤100-day* is likely to be a statistical artifact resulting from genetics and clinical predispositions of donor-source to transplant outcomes; *k* = 1.0037, Panel A in Fig 1. Pre-transplant criterion could have contributed to this phenomenon in related-HSCTs *t≤100-day*.

During MLE, the inclusion of a frailty component was unable to reach a convergence with the use of univariate and multivariate parametric models, and this finding was further verified using a Cox-frailty model. In parametric survival analyses, the convergence of two hazard lines represents the presence of heterogeneity, however, when a multivariate model is introduced and a frailty survival model does not serve any meaningful inference, such hazard convergence suggests a maximum manifestation of senescence, Panel B in Fig 1. If a failed life-saving opportunity is a similar indication of death experienced by patient and no further transplant is offered, an arbitrary right-censored data shall then support this finding. In human population studies, this is the first empirical scenario that such convergence occurs in the absence of frailty.

For advancements made in technology and novel treatments, the final multivariate analysis was considered for periodic effects as the structure of most survival regression models lacks an important dimension; calendar time. Instead of introducing further complexity to the modified likelihood estimation, a biennial event-history analysis (EHA) was introduced to survival profiles of patients with post-transplant survival of *t>365-day*. In conjunction with EHA analysis, the modified likelihood estimation which permits the inclusion of the logical matrix post-transplant survival time-lapse intervals (*t*) completes a three-dimensional analysis; age (*x*, *j*), right-censoring (*θ*) and calendar time (*tx*). This research analysis has shown that multivariate EHA is required to account for biennial medical improvements, if not, the obtained estimates will be misleading (Panel C in Fig 1: Multivariate No EHA and Panel D in Fig 1: Multivariate EHA). Once medical progress has been adjusted for, the hazard lines by donor source suggests that a proportional hazard model shall be valid for HSCTs patients *t>365-day*; Panel D in Fig 1.

## Discussion

### Weibull bends

The human age-specific mortality trajectory of adults in the general population is often assessed using the Gompertzian framework, and the Gompertz hazard function has been considered as the ‘housekeeping’ parametric model for adulthood mortality among demographers and biostatisticians.[17] Under the conditions of extreme immunological responses for patients whom had underwent the removal of their immune cells and had received a new graft from allogeneic donors; *i*.*e*. non-self HSCs, the Weibull function is the most appropriate parametric model to assess their age-specific mortality trajectory, *i*.*e*. the changes in the rate of aging and hazard shape at different post-transplant survival duration; *μ(x) = λ k x*^*(k−1)*^. Parameter *k* is responsible for Weibull’s hazard shape; when *k = 1*, it results to *μ(x) = λ*, a flat hazard. In comparison to Gompertz hazard, Weibull offers a flexibility that the classic Gompertz framework is not able to achieve; Panel C in S1 Fig.

This study has two unexpected findings that merit discussion in the field of aging and biostatistics. Unrelated-HSCTs inverse J-shaped hazard *t≤100-day* suggests a lower immune-reactivity is better for graft-tolerance and survival. It is a scenario whereby the assumption made in antagonistic pleiotropy theory is violated; genetic expressions favoring survival and fitness during mid-adulthood will have a negative effect on longevity at higher ages, Panel A in Fig 1.[18,19] When *t>100-day*, post-transplant patients are characterized by inflammatory responses such as acute GvHD and the increased risk for the onset of chronic GvHD. Proportion of GvHD-types among transplant patients is thereby subjected to changes with increasing survival time; patients with acute GvHD has a higher likelihood of death, and it accounts for >70% of deaths at *t≤100-day*, and all transplant patients who have survived beyond *100 days* are at risk to developing chronic GvHD. As the susceptibility to developing GvHD-types and the demands on cell-mediated immunity change with increasing survival time, it is an analytical error to not categorize survival profiles by post-transplant time-lapse intervals. Hence, the *δ* logical matrix has to be introduced to the parametric likelihood estimation.

At *100-day<t ≤12-month*, the convergence of two hazard lines of a multivariate survival analysis on semi-logarithmic plot is in support of Strehler and Mildvan’s theory; predetermined lifespan in organisms and systems of the body have to recover to its original state of vitality, Panel B in Fig 1.[20] Otherwise, a rapid deterioration in vitality and senescence leading to death. This may be the first empirical study which is based on the survival profiles of transplant patients to present the maximum manifestation of senescence, and this manifestation is shown between related- and unrelated-HSCTs age-specific mortality trajectories.

### The standalone covariate—Year of transplant

In clinical studies, it is a common practice to introduce the year of transplant as a covariate or as a time-interactive covariate during survival analyses. The modified likelihood estimation presented in this study permits the inclusion of both time and age without the necessity for stratification and to avoid correlation of the two time entities. Stratification blinds the effect of the covariate on the risk estimates of interest which may serve its usefulness in some clinical risk-exposure studies. The main difference in the aforementioned modified likelihood approach is to permit a detailed overview of the mortality dynamics at each specific time interval using *δ* as a binary or logical matrix, and it assists in the assessment of clinical interventions and public health audit. Based on the estimates of the no-EHA multivariate and EHA multivariate model, this finding suggests the inclusion of year of transplant as a standalone covariate (no-EHA) is not sufficient to adjust for medical progress made across calendar time; e.g. improvements in transplant protocols, the availability of more effective and safe immunosuppression drugs, better diagnostics tools, pre- and post-transplant care.

### Popularity of arbitrary right-censoring

Arbitrary right-censoring may be a choice of convenience to addressing research hypotheses,[14,15,21,22] but such analytical practice reduces heterogeneity and this data conditioning restricts a better overview of post-transplant survival and disease relapse using multi-state survival regression models, *e*.*g*. Markov Chain, MCMC. As shown in Fig 1, extreme inflammatory and immune-reactivity can alter the rate of aging and age-specific mortality trajectories. In support to previous literature, patients who received related donor-source, a preferred choice in transplant studies, do enjoy a lower likelihood for death. Although the modified likelihood estimation procedure is applied in a survival data that was subjected to arbitrary right-censoring, it can be applied in other data analytical work that contain survival information; age at transplant, age at death, year of transplant, clinical variables, etc.

This study does not justify the importance of age-specific biomarkers that may elevate the risk for disease relapse, poor graft survival and prognosis. These information should be considered for given the data availability to assess the changes in the rate of aging and the modified likelihood estimation can be implemented for such assessment; Equations 1–4.[23,24] It is within the author’s speculation that future multidisciplinary work on age-specific mortality trajectories of transplant patients will improve pre-transplant treatment regimen and a lower mortality rate and rate of aging among first year post-transplant HSCTs patients can be achieved.

## Supporting information

S1 Equation Gompertz-based models. (WMF)

S2 Equation Weibull-based models. (WMF)

S3 Equation Survival function. (WMF)

S1 Fig United States observed mortality rates of males, Year 2000–2005; Absolute plot (Panel A); Semi-logarithmic (Panel B); Gompertz Illustration (Panel C). (PDF)

S2 Fig Density for ages at death; a) ≤100 days only, b) >100 days but within first 365 days, c) >365 days. (TIFF)

S3 Fig Density for post-transplant survival interval, in months. Vertical dotted line indicates 1.5 months.
(TIFF)

S1 Table Parametric univariate models and parameter estimates. (PDF)

S2 Table Multivariate parametric survival model. (PDF)

S3 Table Parameter estimates of finalized multivariate parametric model by donor source. (PDF)

S4 Table Parameter estimates of finalized multivariate parametric model by donor source and adjusted for regions in the United States. (PDF)
