# Supplementary material for: AFT survival model to capture the rate of aging and age-specific mortality trajectories among first-allogeneic hematopoietic stem cells transplant patients
Source: PLoS One. 2018 Mar 2;13(3):e0193287. doi: 10.1371/journal.pone.0193287 (PMC5834196; doi:10.1371/journal.pone.0193287)
Supplement: S1 Fig — (PDF) [file pone.0193287.s004.pdf]

S1 Fig A. United States observed mortality rates of males, Year 2000 – 2005; Absolute plot.

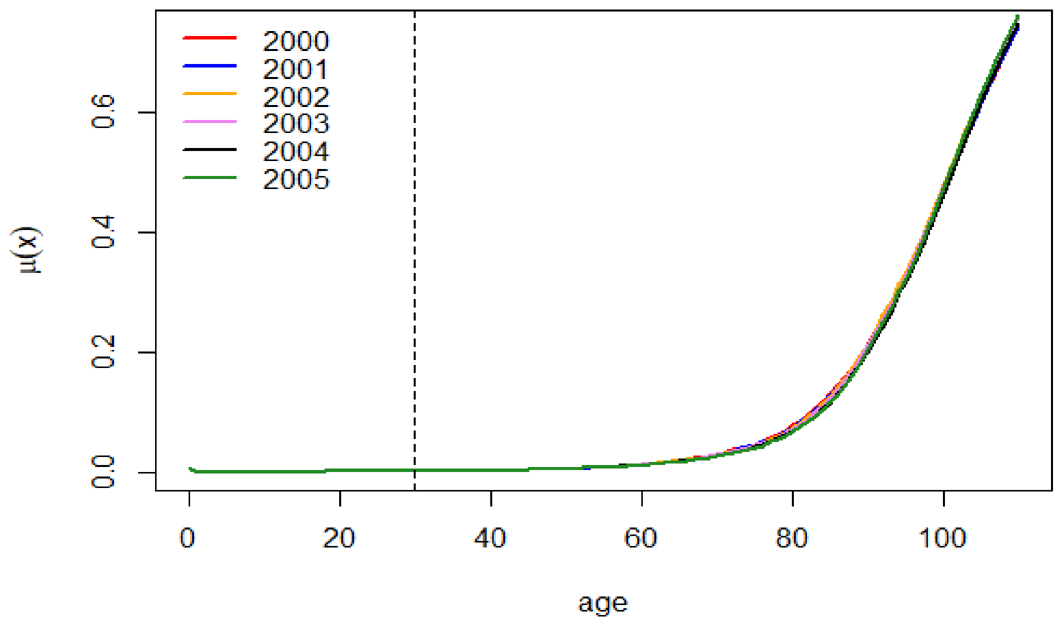

S1 Fig B. United States observed mortality rates of males, Year 2000 - 2005; Semi-logarithmic plot.

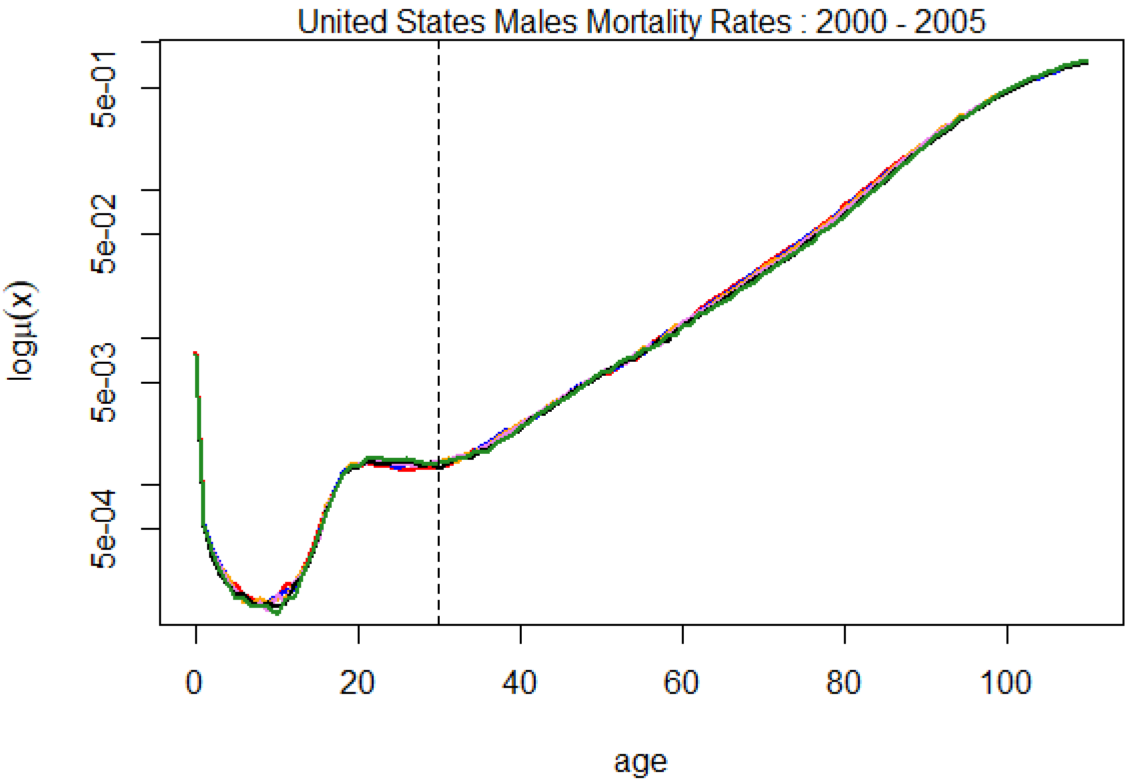

S1 Fig C. A Gompertz Illustration

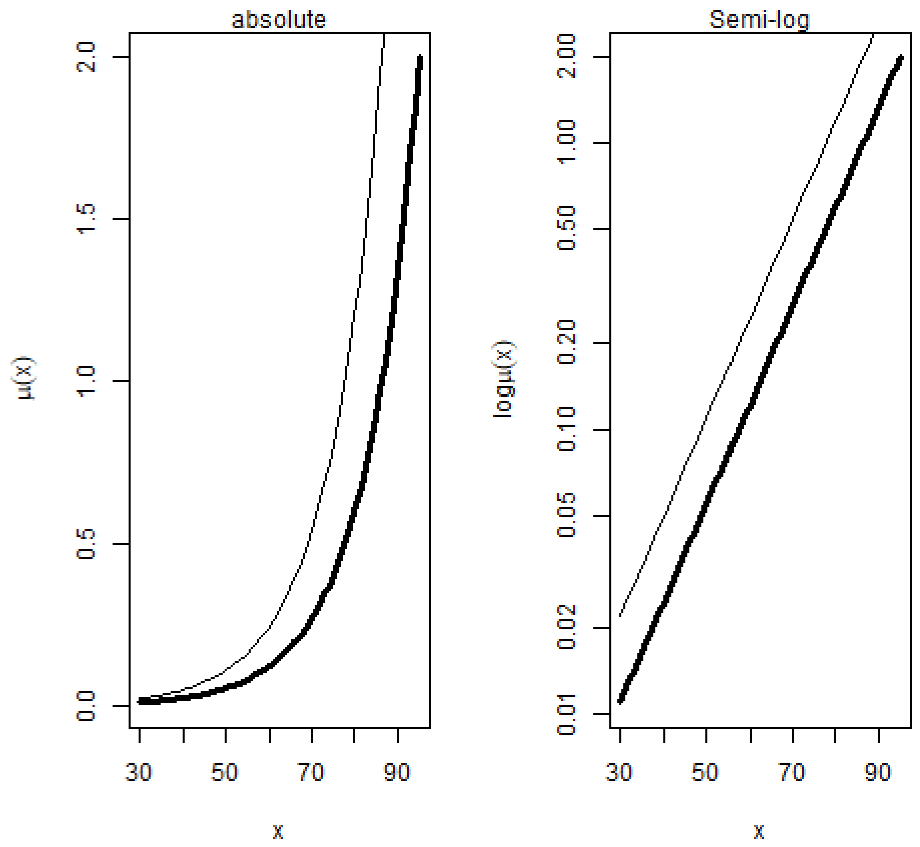
absolute
Semi-log
μ(x)
logμ(x)
0.0
0.5
1.0
1.5
2.0
0.01
0.02
0.05
0.10
0.20
0.50
1.00
2.00
30
50
70
90
x
