# Supplementary figures and images for: AFT survival model to capture the rate of aging and age-specific mortality trajectories among first-allogeneic hematopoietic stem cells transplant patients
Source: PLoS One. 2018 Mar 2;13(3):e0193287. doi: 10.1371/journal.pone.0193287 (PMC5834196; doi:10.1371/journal.pone.0193287)

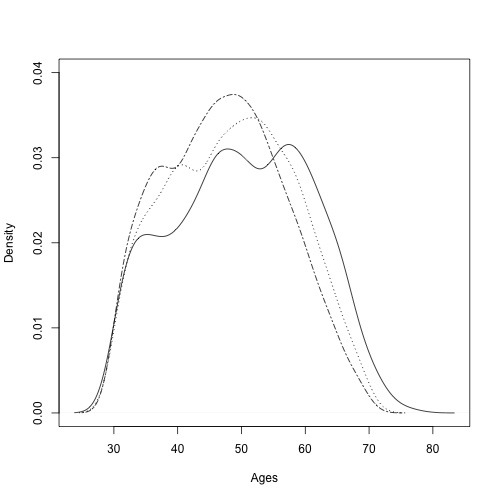

Supplement: S2 Fig — (TIFF) [file pone.0193287.s005.tiff]

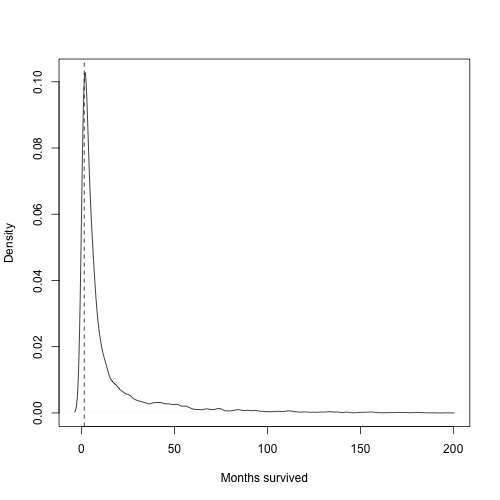

Supplement: S3 Fig — Vertical dotted line indicates 1.5 months. (TIFF) [file pone.0193287.s006.tiff]
