# Supplementary material for: AFT survival model to capture the rate of aging and age-specific mortality trajectories among first-allogeneic hematopoietic stem cells transplant patients
Source: PLoS One. 2018 Mar 2;13(3):e0193287. doi: 10.1371/journal.pone.0193287 (PMC5834196; doi:10.1371/journal.pone.0193287)
Supplement: S1 Table — (PDF) [file pone.0193287.s007.pdf]

S1 Parametric Univariate Models and Parameter Estimates. Parametric models were selected using AIC scores and best fitted model highlighted in bold; Weibull.

Conditions	Parametric Baseline Hazards			
a) ≤ 100 days	*Gompertz*	*Gompertz-Makeham*	*Weibull*	*Weibull-Makeham*
Log-likelihood	*n.c*	*n.c*	***2936.407***	*2936.406*
Parameter estimates				
lambda	-	-	*6.603 (4.697, 9.282)*	*6.232 (0.500, 77.717)*
k	-	-	*1.027 (1.025, 1.028)*	*1.028 (1.026, 1.030)*
c	-	-	-	*0.376 (0.000, U.p.B)*
b) >100days & ≤365days				
Log-likelihood	*n.c*	*606.5486*	***-1049.651***	*-1049.651*
Parameter estimates				
lambda	-	*0.0000 (0.0000, Inf)*	*1.481 (1.374, 1.597)*	*1.480 (1.356, 1.616)*
k	-	*0.1550 (0.0000, U.p.B)*	*1.055 (1.052, 1.058)*	*1.055 (1.052, 1.058)*
c	-	*3.0304 (2.9436, 3.1198)*	-	*0.001 (0.000, Inf)*
c) >365days (N=5,538)				
Log-likelihood	*-7980.363*	*-7980.362*	***-7969.838***	*-7969.838*
Parameter estimates				
lambda	*0.0250 (0.0146, 0.0427)*	*0.0250 (0.0147, 0.0427)*	*0.0017 (0.0001, 0.0427)*	*0.0017 (0.0001, 0.0427)*
k	*0.0265 (0.0157, 0.0450)*	*0.0265 (0.0156, 0.0449)*	*1.8585 (1.7834, 1.9368)*	*1.8584 (1.7832, 1.9367)*
c	-	*0.0000 (0.0000, Inf)*	-	*0.000 (0.000, Inf)*
d) >365days$^{\phi}$				
Log-likelihood	*-7977.121*	*-7977.122*	***-8070.692***	*-7966.176*
Parameter estimates				
lambda	*0.0252 (0.0148, 0.0428)*	*0.0252 (0.0149, 0.0428)*	*0.0018 (0.0001, 0.0420)*	*0.0018 (0.0001, 0.0415)*
k	*0.0266 (0.0157, 0.0450)*	*0.0266 (0.0157, 0.0449)*	*1.8491 (1.7753, 1.9260)*	*1.8494 (1.7759, 1.9260)*
c	-	*0.0000 (0.0000, Inf)*	-	*0.0000 (0.0000, Inf)*

Footnotes:

ϕ: data split by biennial intervals

model selection using AIC

n.c: no convergence

U.p.B: non-infinite values with an upper bound
