# Supplementary material for: AFT survival model to capture the rate of aging and age-specific mortality trajectories among first-allogeneic hematopoietic stem cells transplant patients
Source: PLoS One. 2018 Mar 2;13(3):e0193287. doi: 10.1371/journal.pone.0193287 (PMC5834196; doi:10.1371/journal.pone.0193287)
Supplement: S2 Table — (PDF) [file pone.0193287.s008.pdf]

S2 Multivariate parametric survival model. Weibull as baseline hazard presenting regression estimates and log-likelihoods. ϕ indicates EHA analysis. Further analyses by donor source in Table S3 and geographic regions in Table S4.

Finalized model I: Maximum Likelihood Estimation

	Post-Transplant time lapse			
Weibull	*≤100 days*	*>100 days & ≤365 days*	*>365 days*	*>365days$^{\phi}$*
log-likelihood	*1064.866*	*1239.369*	*-7276.971*	*-10178.44*
Parameter estimates				
lambda	*4.4623 (3.5540, 5.6028)*	*1.1550 (1.0387, 1.2844)*	*0.0083 (0.0006, 0.1161)*	*0.0002 (0.0000, 0.0164)*
k	*0.9986 (0.9986, 0.9987)*	*1.2734 (1.2377, 1.3101)*	*1.5015 (1.4523, 1.5525)*	*1.7219 (1.6583, 1.7880)*
Regression Estimates				
Donor Source (Unrelated)	*1.1882 (1.1762, 1.2002)*	*1.2478 (1.2312, 1.2647)*	*1.7052 (1.6119, 1.8039)*	*1.8583 (1.7485, 1.9750)*
Gender	-	-	*1.1151 (1.1047, 1.1255)*	*1.3063 (1.2764, 1.3369)*
Year of birth	-	1.0211 (1.0209, 1.0213)	-	-
Year of Transplant	*0.9753 (0.9751, 0.9755)*	*0.9592 (0.9587, 0.9597)*	*0.9777 (0.9773, 0.9780)*	-
Agvhd duration, mths	*0.8686 (0.8674, 0.8698)*	*0.8848 (0.8837, 0.8860)*	*0.9906 (0.9906, 0.9906)*	-
Cgvhd duration, mths	-	*0.8540 (0.8514, 0.8566)*	*0.9864 (0.9864, 0.9865)*	*0.9815 (0.9815, 0.9815)*
Waiting time, mths	*1.0038 (1.0036, 1.0039)*	-	-	*0.9150 (0.9102, 0.9199)*
Disease group	*1.0188 (1.0184, 1.0192)*	-	*0.9458 (0.9438, 0.9479)*	-
Prior autologous	-	-	*1.4542 (1.3584, 1.5568)*	*1.1957 (1.1636, 1.2287)*
Total body irradiation	-	-	*1.0228 (1.0224, 1.0233)*	-
Graft types	-	-	*1.3942 (1.3431, 1.4473)*	*2.2616 (2.0976, 2.4384)*
Karnofsky score	-	-	-	*1.1458 (1.1342, 1.1576)*

ϕ: data split by biennial intervals

Covariates that did not significantly improve model fit: conditioning regimen, regigp, disease risk, usregions (not listed)

Optimization method: Simulated Anneal
