# Supplementary material for: AFT survival model to capture the rate of aging and age-specific mortality trajectories among first-allogeneic hematopoietic stem cells transplant patients
Source: PLoS One. 2018 Mar 2;13(3):e0193287. doi: 10.1371/journal.pone.0193287 (PMC5834196; doi:10.1371/journal.pone.0193287)
Supplement: S3 Table — (PDF) [file pone.0193287.s009.pdf]

S3 Parameter estimates of finalized multivariate parametric model by donor source, *i.e.* unrelated vs related. Weibull as baseline hazard and list of covariates shown in Table S2. ϕ indicates EHA analysis.

	**Post-Transplant time lapse**			
Weibull	***≤ 100 days***	***>100 days & ≤ 365 days***	***>365 days***	***>365 days$^{\phi}$***
Unrelated				
lambda	9.6477 (5.3789, 17.3045)	1.1967 (1.0530, 1.3600)	0.0097 (0.0001, 0.9371)	0.0016 (0.0000, 0.1060)
k	1.0308 (1.0291, 1.0326)	1.1335 (1.1162, 1.1511)	1.6134 (1.5034, 1.7314)	1.5359 (1.4765, 1.5978)
log-likelihood	2326.353	-666.2503	-5013.605	-6971.642
Related				
lambda	10.7225 (3.4401, 33.4213)	1.1344 (1.0040, 1.2817)	0.0101 (0.0003, 0.2929)	0.0001 (0.0000, 1.2634)
k	0.9864 (0.9849, 0.9879)	1.1670 (1.1376, 1.1972)	1.4975 (1.4376, 1.5599)	1.8139 (1.6819, 1.9563)
log-likelihood	760.2375	-330.9102	-2271.342	-3201.448
