# Supplementary material for: AFT survival model to capture the rate of aging and age-specific mortality trajectories among first-allogeneic hematopoietic stem cells transplant patients
Source: PLoS One. 2018 Mar 2;13(3):e0193287. doi: 10.1371/journal.pone.0193287 (PMC5834196; doi:10.1371/journal.pone.0193287)
Supplement: S4 Table — (PDF) [file pone.0193287.s010.pdf]

S4 Parameter estimates of the finalized parametric model by donor source and adjusted for regions in the United States. List of clinical covariates (shown in Table S2). Weibull as baseline hazard. ϕ indicates EHA analysis.

	**Post-Transplant time lapse**			
Weibull	***≤100 days***	***>100 days & ≤365 days***	***>365 days***	***>365 days$^{\phi}$***
Unrelated				
lambda	*6.2631 (4.5953, 8.5364)*	*0.7672 (0.5725, 1.0280)*	*0.0139 (0.0022, 0.0882)*	*0.0016 (0.0000, 0.1546)*
k	*0.9613 (0.9599, 0.9627)*	*1.2334 (1.1879, 1.2807)*	*1.6590 (1.6184, 1.7005)*	*1.4842 (1.4236, 1.5474)*
log-likelihood	*1025.613*	*-662.5459*	*-4948.671*	*-6972.142*
Related				
lambda	*5.5735 (4.1923, 7.4097)*	*0.3354 (0.0871, 1.2914)*	*0.0093 (0.0006, 0.1450)*	*0.0003 (0.0000, 1.1865)*
k	*1.0037 (1.0035, 1.0038)*	*1.3964 (1.3144, 1.4836)*	*1.5175 (1.4757, 1.5604)*	*1.4883 (1.4035, 1.5782)*
log-likelihood	*1028.346*	*-327.6726*	*-2299.933*	*-3206.876*
